# Simulation-based feasibility study of monitoring of intracerebral hemorrhages and detection of secondary hemorrhages using electrical impedance tomography

**DOI:** 10.1117/1.JMI.11.1.014502

**Published:** 2024-01-30

**Authors:** Jussi Toivanen, Antti Paldanius, Bachir Dekdouk, Valentina Candiani, Asko Hänninen, Tuomo Savolainen, Daniel Strbian, Nina Forss, Nuutti Hyvönen, Jari Hyttinen, Ville Kolehmainen

**Affiliations:** a University of Eastern Finland, Department of Technical Physics, Kuopio, Finland; b Tampere University, Faculty of Medicine and Health Technology, Tampere, Finland; c University of Genoa, Department of Mathematics, Genoa, Italy; d Helsinki University Hospital, HUS Neurocenter, Helsinki, Finland; e Aalto University, Department of Neuroscience and Biomedical Engineering, Helsinki, Finland; f Aalto University, Department of Mathematics and Systems Analysis, Helsinki, Finland

**Keywords:** brain imaging, electrical impedance tomography, intracerebral hemorrhage, monitoring, stroke

## Abstract

**Purpose:**

We present a simulation-based feasibility study of electrical impedance tomography (EIT) for continuous bedside monitoring of intracerebral hemorrhages (ICH) and detection of secondary hemorrhages.

**Approach:**

We simulated EIT measurements for six different hemorrhage sizes at two different hemorrhage locations using an anatomically detailed computational head model. Using this dataset, we test the ICH monitoring and detection performance of our tailor-made, patient-specific stroke-monitoring algorithm that utilizes a novel combination of nonlinear region-of-interest difference imaging, parallel level sets regularization and a prior-conditioned least squares algorithm. We compare the results of our algorithm to the results of two reference algorithms, a total variation regularized absolute imaging algorithm and a linear difference imaging algorithm.

**Results:**

The tailor-made stroke-monitoring algorithm is capable of indicating smaller changes in the simulated hemorrhages than either of the reference algorithms, indicating better monitoring and detection performance.

**Conclusions:**

Our simulation results from the anatomically detailed head model indicate that EIT equipped with a patient-specific stroke-monitoring algorithm is a promising technology for the unmet clinical need of having a technology for continuous bedside monitoring of brain status of acute stroke patients.

## 1 Introduction

Stroke is the second leading cause of death and a major cause of disability worldwide.^1^ Intracerebral hemorrhage (ICH) accounts for about 20% of all strokes.^1^ ICH is associated with higher mortality than ischemic stroke especially at the early stage.^2^ ICH is typically caused by disruption of cerebral arteries, which leads to bleeding into brain tissue. ICH harms the brain via several different mechanisms,^3^ including the space-occupying mass effect in the acute phase, the toxic effect of the degradation products from the lysed red blood cells, and the inflammatory changes in the later stages. So far, there is no established outcome-modifying medical or surgical treatment for the index ICH. However, one of the major challenges in the acute stage (within 36 h from onset of ICH) is the risk of hematoma expansion and rebleeding.^2^ In such cases, intensive conservative treatment, including controlled mechanical ventilation and optimized blood pressure levels, and timely neurosurgical intervention are likely to be life-saving and even reduce disability. Such intensive care is not risk free, and it should be reserved for patients that show signs of hematoma growth and changes in the level of consciousness. In addition to patients with hemorrhagic stroke, patients with thrombolysis-treated acute ischemic stroke have an increased risk of cerebral hemorrhage as a complication of recanalization therapy. In both patient groups, the decision to advance to intensive care has to be done promptly, and therefore, hematoma expansion or rebleeding in ICH patients should be detected as soon as possible, optimally immediately.

The monitoring of hemorrhagic stroke in the intensive care unit (ICU) is based mainly on routine follow up of clinical signs and symptoms.^2^ However, evaluation of the vigilance of the patient, as well as observing subtle neurological signs can be very challenging, particularly if the patient is sedated and intubated. Currently, the most reliable way to monitor the progression of hemorrhagic stroke is repeated CT scanning^2^ that requires the patient to be repeatedly moved from ICU to CT and back. This is a demanding and time-consuming procedure and can even be dangerous for the patient. Rarely, in some individual stroke centers, moving the patient can be avoided if a bedside head CT scanner is available. Regardless, the selection of correct timing of the control CT imaging is itself already a difficult problem and CT images provide only occasional snapshots of the bleeding. A method for online bedside monitoring of hemorrhagic stroke at the ICU would most likely positively affect patients’ prognosis as there would be no delays in the detection of life-threatening complications and their care.

One potential bedside monitoring method is provided by electrical impedance tomography (EIT). In the EIT measurement setup, small alternating currents are fed through electrodes attached to the patient’s scalp, and the resulting voltages are measured on the electrodes. This safe and radiation-free measurement is then used to compute a three-dimensional image of the electrical conductivity of the brain, with hemorrhage showing as increased intensity with respect to normal brain tissue in the image due to the high conductivity of blood. This type of absolute imaging EIT has been investigated for early differentiation of stroke types, see e.g. Malone et al.,^4^ Goren et al.,^5^ Horesh et al.,^6^ Candiani et al.^7^ for absolute imaging and Agnelli et al.,^8^ McDermott et al.,^9^ Candiani et al.^10^ for machine learning-based approaches. However, EIT-based stroke differentiation is very challenging due to the instability of the absolute imaging EIT problem with respect to modeling errors, such as inexact electrode locations and head shape. Furthermore, for differentiation, a very high sensitivity is required because even very small quantities of blood should not be overlooked in clinical decision making. In a continuous bedside monitoring setup, the EIT measurement is repeated at a later time, and a three-dimensional image of the conductivity change of the brain between the measurements is computed. These types of difference imaging EIT approaches are promising for monitoring of hemorrhagic stroke and detection of secondary hemorrhages as they have good sensitivity, as suggested by simulation studies by Shi et al.,^11^ animal studies by Xu et al.,^12^ and human studies by Dai et al.^13^ and Yang et al.,^14^ and they are less prone to modeling errors.

In the envisioned EIT-based stroke monitoring setup, the patient is admitted to the emergency department of the hospital because of symptoms of acute stroke. According to stroke treatment protocol, a CT scan is taken immediately for diagnosis and timely beginning of suitable treatment. The treatment of the patient continues in the ICU or in a stroke unit, where the EIT measurement device is attached using something akin to an EEG electrode cap. The EIT measurements, taken, e.g., every 2 to 10 min, and an imaging algorithm are then used to get snapshots of the conductivity changes in the brain, indicative of changes in the hemorrhage, which would indicate any immediate need for confirmatory CT imaging or medical intervention. The EIT-based stroke imaging could also be used in a similar manner for monitoring of occurrence of secondary hemorrhages in treatment of ischemic stroke patients. Furthermore, the CT image routinely taken at patient admission can be utilized for generation of a 3D computational model of the patient’s head and for regularization purposes in the image reconstruction.

In this paper, we study the feasibility of EIT for monitoring and detection of ICHs with simulated measurement data from an anatomically highly detailed head model. We compare the performance of several inverse estimation methods for the monitoring task. We use the algorithm first introduced by Toivanen et al.^15^ that has been constructed as a combination of the nonlinear region-of-interest difference imaging approach by Liu et al.^16^ and the parallel level sets regularization approach by Kolehmainen et al.^17^ In addition, it now also utilizes a prior-conditioned least squares algorithm, see Arridge et al.^18^ and Harhanen et al.,^19^ for computationally efficient solution of the lagged Gauss–Newton search direction for the minimization of the regularized nonlinear least squares functional. Furthermore, a more advanced initial estimation approach is used. We use an atomically detailed six-layered head model with intricate cerebrospinal fluid and brain geometry that was first introduced by Paldanius et al.^20^ to simulate EIT measurements with various ICH locations and volumes to test our stroke monitoring and detection algorithm. The results with our new monitoring algorithm are compared to the results of two reference algorithms, a total variation (TV) regularized absolute imaging algorithm and a linear difference (LD) imaging algorithm.

### 2 Theory

#### 2.1 Modeling of EIT Measurements

We model the patient’s head in the EIT measurement setup as a domain $\Omega \subset R^{3}$, and the L electrodes attached to its surface with circular surface patches $e_{\ell }$, l=1,2,…,L. During the measurement, P patterns of currents, $I^{\left(k\right)}\in R^{L}$, k=1,2,…,P, are consecutively injected through the electrodes, and the corresponding voltages $U^{\left(k\right)}\in R^{L}$ are measured on all electrodes. Here $I_{\ell }^{\left(k\right)}$ and $U_{\ell }^{\left(k\right)}$ denote the applied current and measured voltage from the k’th current pattern on the ℓ’th electrode for ℓ=1,2,…,L. Based on the conservation of charge and our choice of electric potential ground, we have

$$\overset{L}{\underset{\ell =1}{\sum }}I_{\ell }^{\left(k\right)}=0\;\overset{L}{\underset{\ell =1}{\sum }}U_{\ell }^{\left(k\right)}=0.$$ (1)

The voltages $U_{\ell }^{\left(k\right)}$ are boundary measurements of the interior electromagnetic potential $u^{\left(k\right)}(x)$ that is modeled with the conductivity equation

$$\nabla \cdot (\sigma (x)\nabla u^{\left(k\right)}(x))=0,\;x\in \Omega$$ (2)

and the boundary conditions of the complete electrode model (CEM)^21^^,^^22^ for k=1,…,P and ℓ=1,…,L

$$u^{\left(k\right)}(x)+z_{\ell }\sigma (x)\frac{\partial u^{\left(k\right)}(x)}{\partial n}=U_{\ell }^{\left(k\right)},\;x\in e_{\ell },$$ (3)

$$\int_{e_{\ell }}\sigma (x)\frac{\partial u^{\left(k\right)}(x)}{\partial n}dS=I_{\ell }^{\left(k\right)},$$ (4)

$$\sigma (x)\frac{\partial u^{\left(k\right)}(x)}{\partial n}=0,\;x\in \partial \Omega \setminus \overset{L}{\underset{\ell =1}{⋃}}e_{\ell },$$ (5)

where $z_{\ell }$ is the contact impedance between the electrode $e_{\ell }$ and the body Ω, n denotes the outward unit normal vector on the boundary ∂Ω, and the isotropic conductivity distribution σ is assumed to belong to $L_{+}^{\infty }(\Omega )≔\{\varsigma \in L^{\infty }(\Omega )|\mathrm{ess}\;\inf\;\varsigma >0\}$. The existence and uniqueness of the solution $(u,U)\in H^{1}(\Omega )⊕R^{L}/R$ of the model [Eqs. (1)–(5)] were proven and its variational form derived by Somersalo et al.^22^

In this paper, for the imaging algorithms, the numerical solution of the model [Eqs. (1)–(5)] is based on the finite-element method (FEM), for details of the implementation see Vauhkonen et al.^23^ and Kaipio et al.^24^ In the following, we denote the FEM-based solution for a single current pattern $I^{\left(k\right)}\in R^{L}$ by $\sigma \mapsto U(\sigma ;I^{\left(k\right)})\in R^{L}$ where we consider a discretized version of σ, such that $\sigma =\overset{N}{\underset{j=1}{\sum }}\sigma_{j}\varphi_{j}$, where $\sigma_{j}\in R_{+}$ are the nodal coefficients, j=1,…,N, and $\varphi_{j}\in H^{1}(\Omega )$, j=1,…,N, are the piecewise linear basis functions corresponding to an FE mesh of Ω. Similarly for other variables, we use identical notation for continuous and discretized forms, assuming the correct interpretation is clear from the context.

The measurement noise $e\in R^{P\cdot L}$ is modeled as additive noise, leading to the measurement model

V=U(σ)+e, (6)

where $V\in R^{P\cdot L}$ is the vector of the measured noisy voltages for all applied current patterns and $U(\sigma )={\left(U(\sigma ;I^{\left(1\right)}),\ldots ,U(\sigma ;I^{\left(P\right)})\right)}^{T}\in R^{P\cdot L}$.

#### 2.2 Algorithms

In EIT stroke monitoring, the occurrence of a secondary hemorrhage during treatment of ischemic stroke or the growth of a hemorrhagic stroke is detected as a change in conductivity

$$\delta \sigma =\sigma_{2}-\sigma_{1}$$ (7)

between two consecutive measurement times $t_{1}$ and $t_{2}$. Because the hemorrhage-related changes are slow compared to the duration of a single EIT measurement set, it is reasonable to model the measurements at $t_{1}$ and $t_{2}$ with the stationary model Eq. (6) as

$$V_{1}=U(\sigma_{1})+e_{1},\;V_{2}=U(\sigma_{2})+e_{2}.$$ (8)

With this setup, we test the feasibility of EIT for monitoring and detection of ICH by comparing the performance of three algorithms for obtaining δσ as follows.

(1) A total variation (TV) regularized absolute imaging algorithm that reconstructs $\sigma_{1}$ and $\sigma_{2}$ independently and obtains δσ by subtraction.
(2) A linear difference imaging algorithm (LD) that directly reconstructs δσ.
(3) A stroke-monitoring algorithm (MO) that reconstructs $\sigma_{1}$ and δσ utilizing a novel combination of nonlinear region-of-interest difference imaging,^16^ parallel level sets regularization,^17^ and a prior-conditioned least squares algorithm for solution of the lagged Gauss–Newton search direction in the minimization of the regularized nonlinear least squares functional.^18^^,^^19^

##### 2.2.1 Absolute imaging-based algorithm

In the TV regularized absolute imaging algorithm, δσ is obtained by first solving two separate absolute imaging problems to obtain estimates of $\sigma_{1}$ and $\sigma_{2}$ and then computing $\delta \sigma =\sigma_{2}-\sigma_{1}$. The absolute imaging problem is solved with one of the most popular reconstruction methods, the generalized Tikhonov regularization:

σ^t=arg minσt>0{‖Le(V−U(σt))‖2+pσ(σt)}, (9)

where t=1,2, $L_{e}$ is a Cholesky factor of the noise precision matrix, i.e., $L_{e}^{T}L_{e}=\Gamma_{e}^{-1}$, and $p_{\sigma }(\sigma_{t})$ is a regularization functional, which is in this algorithm chosen as smoothed TV regularization^25^

$$p_{\sigma }(\sigma_{t})=\mathrm{TV}(\sigma_{t})=\alpha \int_{\Omega }{\left({\left\|\nabla \sigma_{t}\right\|}^{2}+\beta^{2}\right)}^{1/2}dx,$$ (10)

where α>0 is the regularization weight coefficient, ∇σ is the gradient of the conductivity σ, and β>0 is a small smoothing parameter that ensures differentiability. The minimization problem [Eq. (9)] is solved with a lagged Gauss–Newton method equipped with a line search algorithm that also enforces the nonnegativity σ>0. To obtain a starting point $\sigma_{t}^{\left(0\right)}$ for the iteration, the best fitting constant conductivity can be estimated by solving a nonlinear least squares fitting problem.

Although the absolute imaging approach is generally very sensitive to geometric modeling errors, it can be expected to be a feasible choice in the stroke monitoring setup, where the patient-specific head geometry is available from the patient CT taken for diagnosis of the stroke and the geometry of the domain does not change during the monitoring.

##### 2.2.2 Linear difference imaging algorithm

In linear difference imaging, see Barber et al.^26^ and Bagshaw et al.,^27^ the aim is to reconstruct the change in conductivity between measurements, $V_{1}$ and $V_{2}$. In the linear difference imaging algorithm of this paper, the measurement models [Eq. (8)] are linearized at some conductivity $\sigma_{0}$ using the first-order Taylor approximations

$$V_{1}\approx U(\sigma_{0})+J(\sigma_{1}-\sigma_{0})+e_{1},$$ (11)

$$V_{2}\approx U(\sigma_{0})+J(\sigma_{2}-\sigma_{0})+e_{2},$$ (12)

where the Jacobian matrix J is evaluated at $\sigma_{0}$. A reasonable choice for the linearization point $\sigma_{0}$ can be obtained by solving a nonlinear least squares fitting problem for the best fitting constant conductivity using the measurement data $V_{1}$. With the linearized models Eqs. (11) and (12), the difference in measurements can be written as

$$\delta V=V_{2}-V_{1}=(U(\sigma_{0})+J(\sigma_{2}-\sigma_{0})+e_{2})-(U(\sigma_{0})+J(\sigma_{1}-\sigma_{0})+e_{1})=J\delta \sigma +\delta e,$$ (13)

where $\delta \sigma =\sigma_{2}-\sigma_{1}$ and $\delta e=e_{2}-e_{1}$. The linear difference imaging problem is to reconstruct δσ based on the difference data δV and the solution can be obtained in the form of the generalized Tikhonov solution [Eq. (9)] as

δ^σ=arg minδσ{‖Lδe(δV−Jδσ)‖2+pLD(δσ)}, (14)

where $L_{\delta e}$ is the Cholesky factor of the noise precision matrix of δe so that $L_{\delta e}^{T}L_{\delta e}=\Gamma_{\delta e}^{-1}=(\Gamma_{e_{1}}+\Gamma_{e_{2}}{)}^{-1}$. The linear regularization functional $p_{\mathrm{LD}}(\delta \sigma )$ is in this paper a smoothness regularization implemented utilizing a distance-based correlation model, giving

$$p_{\mathrm{LD}}(\delta \sigma )={\left\|L_{p}\sigma \right\|}^{2},$$ (15)

so that $L_{p}^{T}L_{p}=\Gamma_{p}^{-1}$ where the covariance matrix $\Gamma_{p}$ is constructed using distance-based correlation:^28^

$$\Gamma_{p}(i,j)=\mathrm{std}{\left(\sigma \right)}^{2}\;\exp(-\frac{{\left\|x_{i}-x_{j}\right\|}^{2}}{2a^{2}}),$$ (16)

where i,j=1,…,N, and the parameter a controls the correlation length and can be solved by setting the distance $\left\|x_{i}-x_{j}\right\|$ to a selected value d (e.g., half the radius of the target) and setting $\Gamma_{p}(i,j)$ to the desired covariance for that distance (e.g., 1% of the variance).

One of the main reasons for the popularity of linear difference imaging is that in the difference measurements [Eq. (13)] usually at least part of the systematic modeling errors are cancelled out, making the approach more tolerant to modeling errors compared to most absolute imaging-based approaches. However, as the approach is based on a linear approximation of the nonlinear problem, it can lead to suboptimal results if the change between the states is large or, as usually in practice, the initial state is unknown, implying that $\sigma_{0}\neq \sigma_{1}$ and the linearization is carried out in a nonoptimal point.

##### 2.2.3 Stroke-monitoring algorithm

The stroke-monitoring algorithm has been constructed as a novel combination of the nonlinear region-of-interest difference imaging approach by Liu et al.^16^ with the parallel level sets regularization approach by Kolehmainen et al.^17^ and it utilizes a prior-conditioned least squares algorithm similar to Arridge et al.^18^ and Harhanen et al.^19^ for computationally efficient solution of the lagged Gauss–Newton search direction in the minimization of the regularized nonlinear least squares problem.

The nonlinear difference imaging approach has been shown to be tolerant to modeling errors to at least the same extent as the linear difference imaging approach and it can produce quantitatively more accurate reconstructions of the conductivity change.^29^^,^^30^ This results from solving the full nonlinear EIT problem instead of the linearized one and from the parameterization of the problem with respect the initial conductivity state and the conductivity change. With this parameterization, the effects of the modeling errors that are invariant between the measurements $V_{1}$ and $V_{2}$ were found by Liu et al.^30^ to induce errors in the reconstruction of the initial state $\sigma_{1}$, leaving the reconstruction of the conductivity change unaffected. Furthermore, in nonlinear difference imaging, it is possible to employ an *a priori* information-based region of interest (ROI) constraint for the conductivity change δσ so that

$$\mathrm{supp}(\delta \sigma )=\Omega_{\mathrm{ROI}}\subseteq \Omega .$$ (17)

In monitoring of ICH a natural, not too constrictive ROI is the brain volume. If no ROI is desired, it is possible to use $\Omega_{\mathrm{ROI}}=\Omega$, but restricting the ROI to a smaller subdomain has been shown to improve the reconstruction of δσ.^16^ Based on Eq. (17), the conductivity at the later measurement time $t_{2}$ is modeled as

$$\sigma_{2}=\sigma_{1}+K\delta \sigma ,$$ (18)

where K is an extension mapping that extends the conductivity change from the ROI to the whole domain Ω, so that

$$K\delta \sigma =\{\begin{array}{ll} \delta \sigma , & x\in \Omega_{\mathrm{ROI}}\\ 0, & x\in \Omega \setminus \Omega_{\mathrm{ROI}}. \end{array}$$ (19)

For the simultaneous estimation of $\sigma_{1}$ and δσ, the measurement data $V_{1}$ and $V_{2}$ are combined into a single vector, leading to the measurement model

$$[\begin{array}{c} V_{1}\\ V_{2} \end{array}]=[\begin{array}{c} U(\sigma_{1})\\ U(\sigma_{1}+K\delta \sigma ) \end{array}]+[\begin{array}{c} e_{1}\\ e_{2} \end{array}].$$ (20)

This measurement model can be written as

$$\tilde{V}=\tilde{U}(\tilde{\sigma })+\tilde{e},$$ (21)

where

$$\overset{˜}{V}=[\begin{array}{c} V_{1}\\ V_{2} \end{array}],\;\overset{˜}{U}=[\begin{array}{c} U(\sigma_{1})\\ U(\sigma_{1}+K\delta \sigma ) \end{array}],$$ (22)

$$\overset{˜}{\sigma }=[\begin{array}{c} \sigma_{1}\\ \delta \sigma \end{array}],\;\overset{˜}{e}=[\begin{array}{c} e_{1}\\ e_{2} \end{array}],$$ (23)

and being now in a form similar to the measurement model Eq. (6), the joint reconstruction of $\sigma_{1}$ and δ can be defined in the form of the generalized Tikhonov regularization [Eq. (9)] as

$$\overset{˜}{\sigma }=\arg\;\underset{\overset{˜}{\sigma }}{\min}\{{\left\|{\overset{˜}{L}}_{e}(\overset{˜}{V}-\overset{˜}{U}(\overset{˜}{\sigma }))\right\|}^{2}+p(\overset{˜}{\sigma })\},$$ (24)

where the diagonal blocks of ${\tilde{L}}_{e}$ contain the Cholesky factors of the noise precision matrices of measurements $V_{1}$ and $V_{2}$, and the regularization functional

$$p(\tilde{\sigma })=p_{\sigma_{1}}(\sigma_{1})+p_{\delta \sigma }(\delta \sigma )$$ (25)

allows independent regularization models for δσ and $\sigma_{1}$. The conductivity change caused by stroke expansion is expected to be localized and regular, and thus we use the smoothed TV regularization^25^

$$p_{\delta \sigma }(\delta \sigma )=\mathrm{TV}(\delta \sigma )=\alpha_{\delta \sigma }\int_{\Omega }{\left({\left\|\nabla \delta \sigma \right\|}^{2}+\beta^{2}\right)}^{1/2}dx,$$ (26)

where $\alpha_{\delta \sigma }>0$ is the regularization weight coefficient, ∇δσ is the gradient of the conductivity change, and β>0 is a small smoothing parameter that ensures differentiability. The initial conductivity $\sigma_{1}$ is expected to correlate well with the structure of the patient CT that is always taken for diagnosis of the stroke at the time of patient admission to the hospital. This information can be utilized using a parallel level sets-based, spatially and directionally weighted TV regularization that promotes similar alignment of level sets in $\sigma_{1}$ and the CT-based reference image,^17^ giving

$$p_{\sigma_{1}}(\sigma_{1})=\mathrm{WTV}(\sigma_{1})=\alpha_{\sigma_{1}}\int_{\Omega }{\left({\left\|\nabla \sigma_{1}\right\|}_{B(\kappa )}^{2}+\beta^{2}\right)}^{1/2}dx,$$ (27)

where $\alpha_{\sigma_{1}}>0$ is the regularization weight coefficient, κ(x) is the reference image and the tensor B(κ) is chosen such that aligned edges (or level sets) in $\sigma_{1}(x)$ and the reference image κ(x) are preferred. One possible choice for the weighting tensor is^17^

$$B(\kappa )=I-(1-\gamma (x))\hat{\nu }(x)\hat{\nu }(x{)}^{T},$$ (28)

where

ν^(x)={0,if  ‖∇κ(x)‖=0∇κ(x)/‖∇κ(x)‖,otherwise, (29)

and 0≤γ(x)≤1 is an edge indicator function such that γ(x)→1 when ‖∇κ(x)‖→0. In this paper, γ(x) is chosen by thresholding the gradient of the reference image so that

$$\gamma (x)=\{\begin{array}{ll} \gamma_{1}, & \text{if}\;\|\nabla \kappa (x)\|\geq \text{threshold}\\ 1, & \text{otherwise}, \end{array}$$ (30)

where $\gamma_{1}\ll 1$.

The minimization problem [Eq. (24)] can be solved using a lagged Gauss–Newton method with the positivity constraints $\sigma_{1}>0$ and $\sigma_{1}+K\delta \sigma >0$. However, solving of the Gauss–Newton search direction is very memory intensive and slow given the large number of unknowns and voltage measurements in the stroke-monitoring setup. In this paper, the search direction is solved in a less memory-intensive way using a prior-conditioned LSQR (MLSQR) iteration.^18^ This iterative method significantly reduces the required number of iterations by limiting its solution space based on the regularization used in the inverse problem. The formulation for prior conditioning also produces an alternative way for obtaining the gradients and Hessians of the regularization terms that are required for the lagged Gauss–Newton iteration (for more details, see Arridge et al.^18^ and Harhanen et al.^19^).

To obtain a good initialization for the optimization of Eq. (24), we utilize an anatomically guided initial estimate where the domain is divided to three subvolumes (scalp, skull, and brain) based on the reference image κ(x) and a three parameter nonlinear least squares estimation is carried out to obtain an initial conductivity with a constant conductivity value for each of the subvolumes.

## 3 Methods

### 3.1 Computational Head Model

**Fig. 1:**
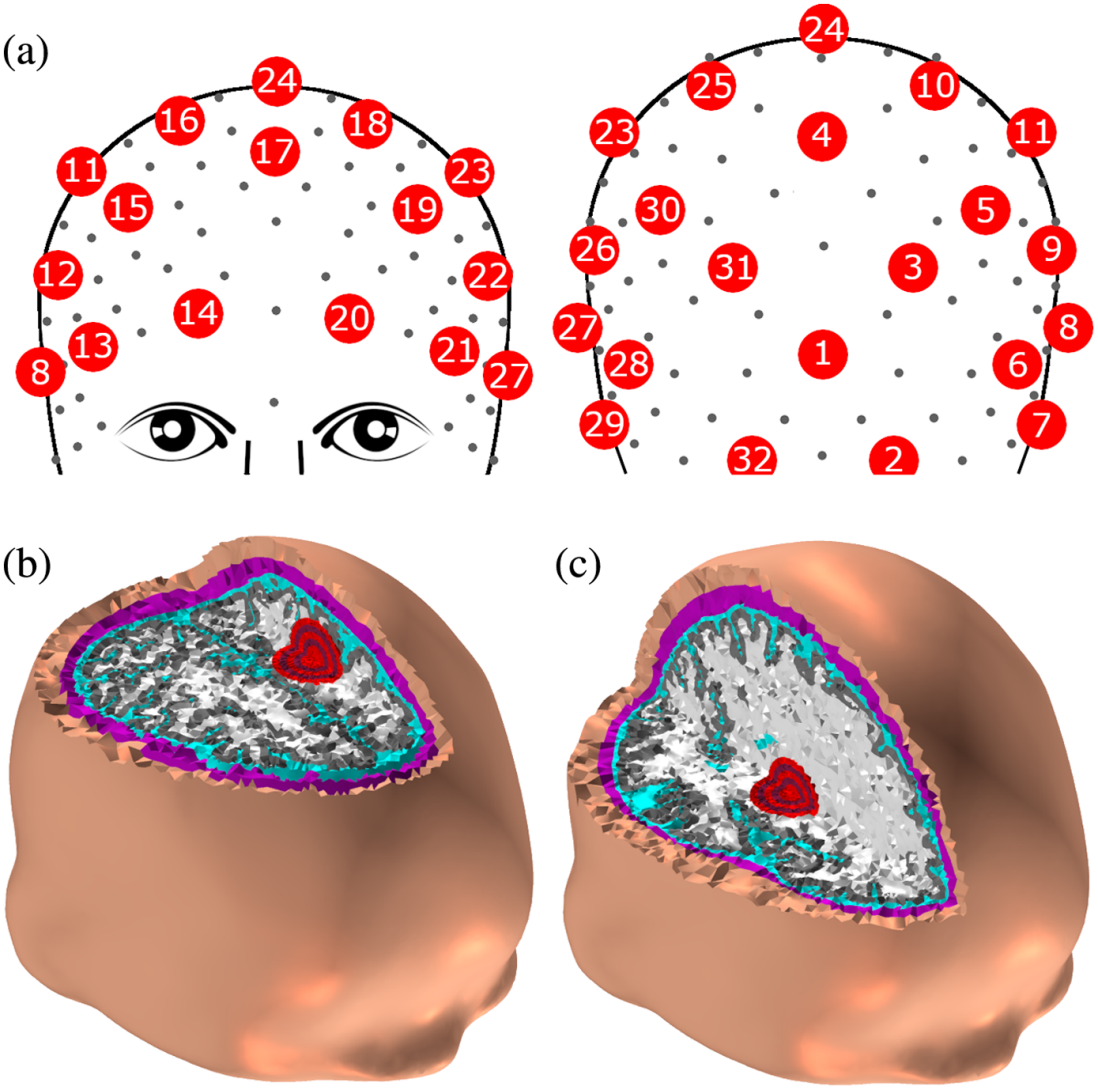
(a) Electrode locations chosen from the 10-5 system as described by Ref. 5. (b) The hemorrhage location close to the surface of the brain. (c) The hemorrhage location deep in the basal ganglia region.

The highly accurate head model used for simulations of the EIT measurements was published by Paldanius et al.^20^ and is based on a model from the population head model repository.^31^ The original head model consisted of surface mesh presentations of the scalp, skull, CSF, white matter, gray matter, and cerebellum. For 3D meshing, these surface meshes were imported into ScanIP Simpleware. 32 circular electrodes with a 10 mm diameter were assigned based on the modified 10-5 EEG electrode placement system designed for EIT measurements [Fig. 1(a)] by Goren et al.^5^ To simulate a hemorrhage growing over time, concentric spheres with diameters from 10 to 30 mm in 5 mm increments were manually placed in the volume of interest in the brain parenchyma. The 30 mm sphere has a volume of 14.14 ml, matching the 14 ml median size of ICH.^32^ For the meshing, the target maximum error from the original surface mesh was set to 0.15 mm to ensure preservation of thin details, such as the layer of CSF around the brain. The maximum element edge length on the electrodes was set to 1 mm to make the mesh denser in the regions near the electrodes as is required for sufficient numerical accuracy in EIT simulations. These settings resulted in a 3D mesh of 2.5 million tetrahedral elements, which was then exported to COMSOL for simulation of the measurement data.

Two locations of simulated hemorrhage were considered, one cortical hemorrhage close to the surface of the brain and one deep in the basal ganglia region of the brain. This provided varying level of challenge for the EIT algorithms, as the sensitivity of the EIT measurement is lower when the perturbation is deeper in the brain parenchyma.^20^ The basal ganglia region is also the most common location of ICH, as perforative small arteries are prone to rupture in patients with high blood pressure. The chosen hemorrhage locations are shown in Figs. 1(b) and 1(c).

Model setup for the simulation was performed in COMSOL 5.6. The tissue layers were assigned with corresponding dielectric material properties from Gabriel et al.^33^ (Table 1). The properties did not include values for the scalp as it is composed of multiple different tissues. Hence, values of muscle tissue which were within the range of values reported for the scalp in other literature^34^ were used. The dielectric values for cortical bone were used for the skull as it matches the recommended value for single-layer skull models.^34^ The material properties for 1 kHz frequency were used, as the measurements were simulated at that frequency.

**Table 1:** Material properties used in the simulation at 1 kHz frequency.^33^

Tissue	Conductivity (S/m)	Permittivity (F/m)
Scalp	0.32	434,932
Skull	0.02	2702
CSF	2.00	109
White matter	0.06	69,810
Gray matter	0.10	164,062
Cerebellum	0.12	164,358
Blood	0.70	5259

As COMSOL does not natively support the CEM, the CEM implementation tailor-made for COMSOL from Fouchard et al.^35^ was used. An iterative BiCGStab solver was selected for solving the FEM system.

The growth of the hemorrhage was simulated by changing the material properties of the concentric spheres representing the hemorrhage from white matter and gray matter to blood. In the first simulation, all the spheres were assigned material properties of white matter for the hemorrhage close to surface of the brain and gray matter for the hemorrhage deep in the basal ganglia region to represent a healthy brain. In the following simulations, the spheres were sequentially assigned material properties of blood with the hemorrhage finally being 30 mm in diameter in the last simulation.

### 3.2 Simulated Measurement Setup

In the simulation, a total of 32 independent pairwise current injections at 1 kHz and 1 mA magnitude were used. The injection pattern used was 1-13, 2-14,…, 32-12, where the first number is the number of the current injecting electrode and the second number is the current sink electrode. In this current injection pattern, there is sufficient distance between the current injecting and current sink electrodes for some of the current to pass through the skull and the brain, instead of using adjacent electrodes resulting in current being mainly shunted along the well conducting scalp. For each injection, the noninjecting electrodes recorded the resulting potentials from the scalp. The simulated measurement data were saved as differential potentials between electrodes 1-2, 2-3,…, 32-1 and exported in .csv format.

Noisy realizations of the simulated measurement data were obtained by adding Gaussian zero mean random noise with a standard deviation of $1.84\times 10^{-5}\;V$ to the simulated noise-free measurements. The standard deviation of the noise was 0.067% of the maximum amplitude of the EIT data from a healthy brain and corresponds to the approximated relative noise level of the prototype stroke measurement device from Toivanen et al.^15^

### 3.3 Computational Meshes and Regularization Parameters for the Imaging Algorithms

**Fig. 2:**
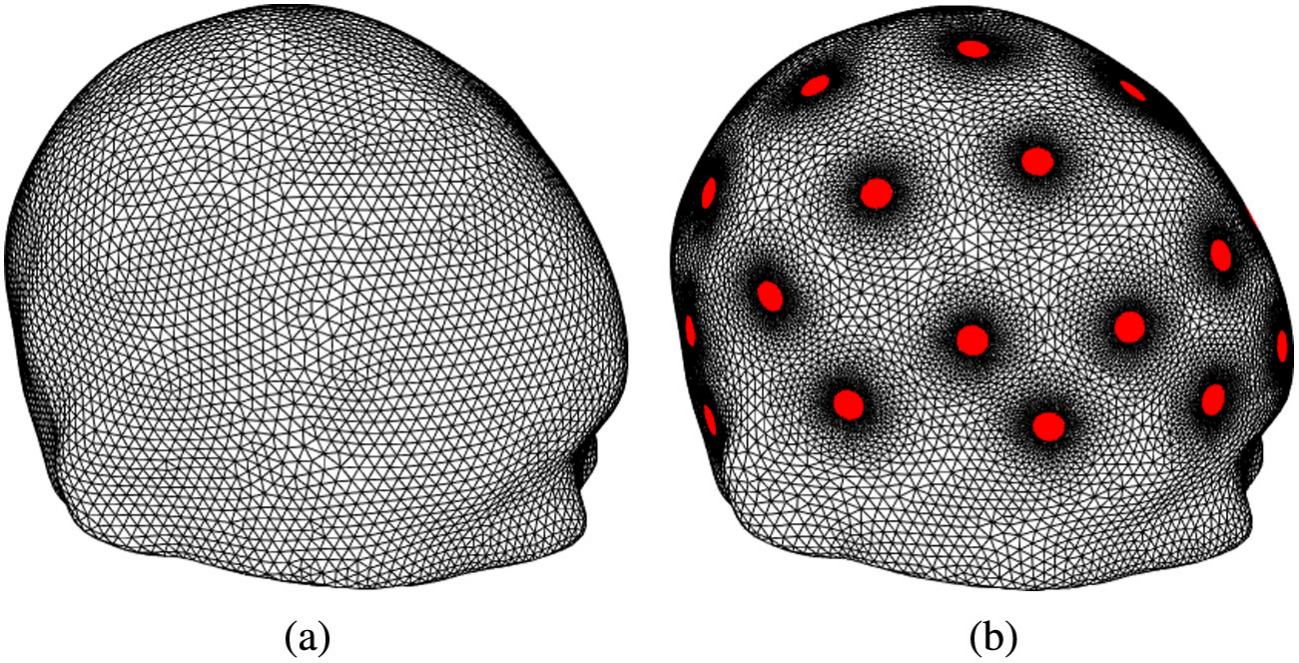
Computational meshes used for the imaging algorithms. The computational mesh for (a) conductivity and (b) the electric potential with electrodes highlighted.

All imaging algorithms used a MATLAB-based solver for the FEM model [Eq. (6)] where the electric potential u(x) was approximated in a tetrahedral mesh of 116,235 nodes and 597,631 elements with refinement near the electrodes as is required for sufficient accuracy for the numerical solving of the model [Eqs. (1)–(5)]. Such refinement is not needed for the discretization of the conductivity and would unnecessarily increase the number of unknown conductivity values. Thus the conductivity was approximated in a coarser and uniform tetrahedral mesh of 38,433 nodes and 207,453 elements, leading to N=38,433 unknown conductivity values. These meshes used for the imaging algorithms are shown in Fig. 2 and they have a considerably smaller number of elements than the highly accurate, 2.5 million element mesh used in COMSOL for measurement data simulation from the anatomically detailed head model.

**Fig. 3:**
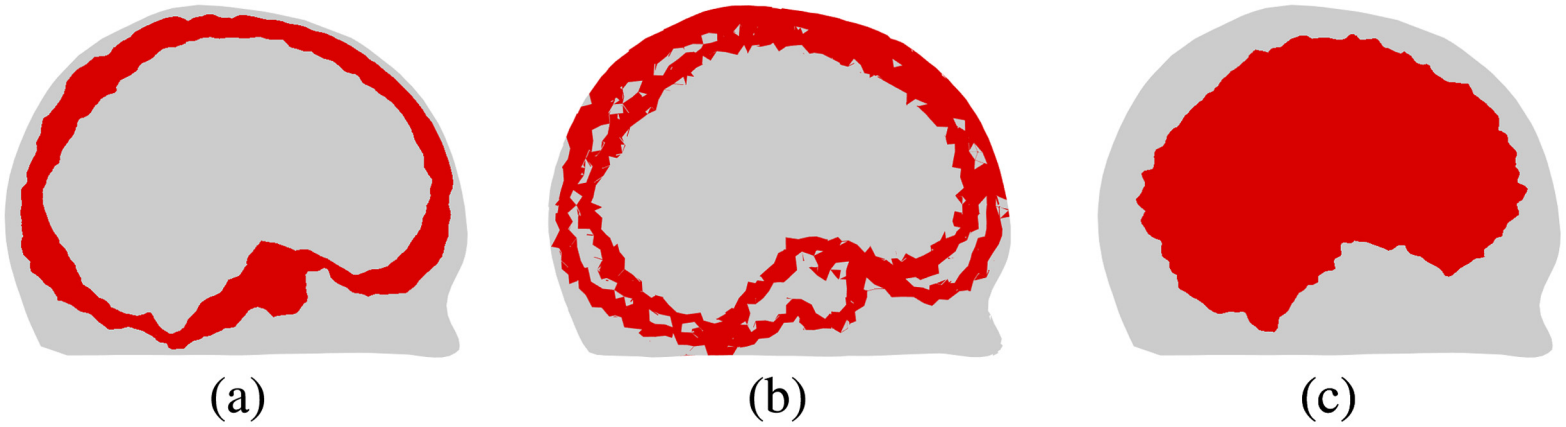
(a) Cross sections of the reference image κ(x), (b) the indicator function γ(x), and (c) the region of interest used for the stroke-monitoring algorithm.

The regularization parameters for each algorithm were tuned manually to give the best visual quality of the reconstructions. For the TV regularized absolute imaging algorithm, the values α=0.01 and β=0.001 were used in Eq. (10). For the linear difference imaging algorithm, a standard deviation of conductivity $\mathrm{std}(\sigma )=2\sigma_{0}$ was used in Eq. (16) and the parameter a was calculated by setting a covariance of 1% of the variance at a correlation distance $d=l_{\Omega }/4$, where $l_{\Omega }=20.4\;\mathrm{cm}$ is the length of the domain Ω from the back of the head to the forehead. For the stroke-monitoring algorithm, the values $\alpha_{\delta \sigma }=0.005$ and β=0.001 were used in Eq. (26), the values $\alpha_{\sigma_{1}}=10^{-7}$ and β=0.001 were used in Eq. (27), and cross sections of the reference image κ(x), the indicator function γ(x) and the region of interest are shown in Fig. 3. The reference image was obtained by approximately mapping the true skull volume into the computational mesh used for discretization of the conductivity, in a similar manner as a CT image could be used in a clinical setup. The indicator function γ(x) corresponds to the boundaries in the reference image and the region of interest was chosen to approximately correspond to the brain volume. This would be a safe choice also when monitoring for secondary hemorrhages, where the hemorrhage can sometimes occur also outside of the brain volume weakened by the ischemic stroke.

## 4 Results and Discussion

**Fig. 4:**
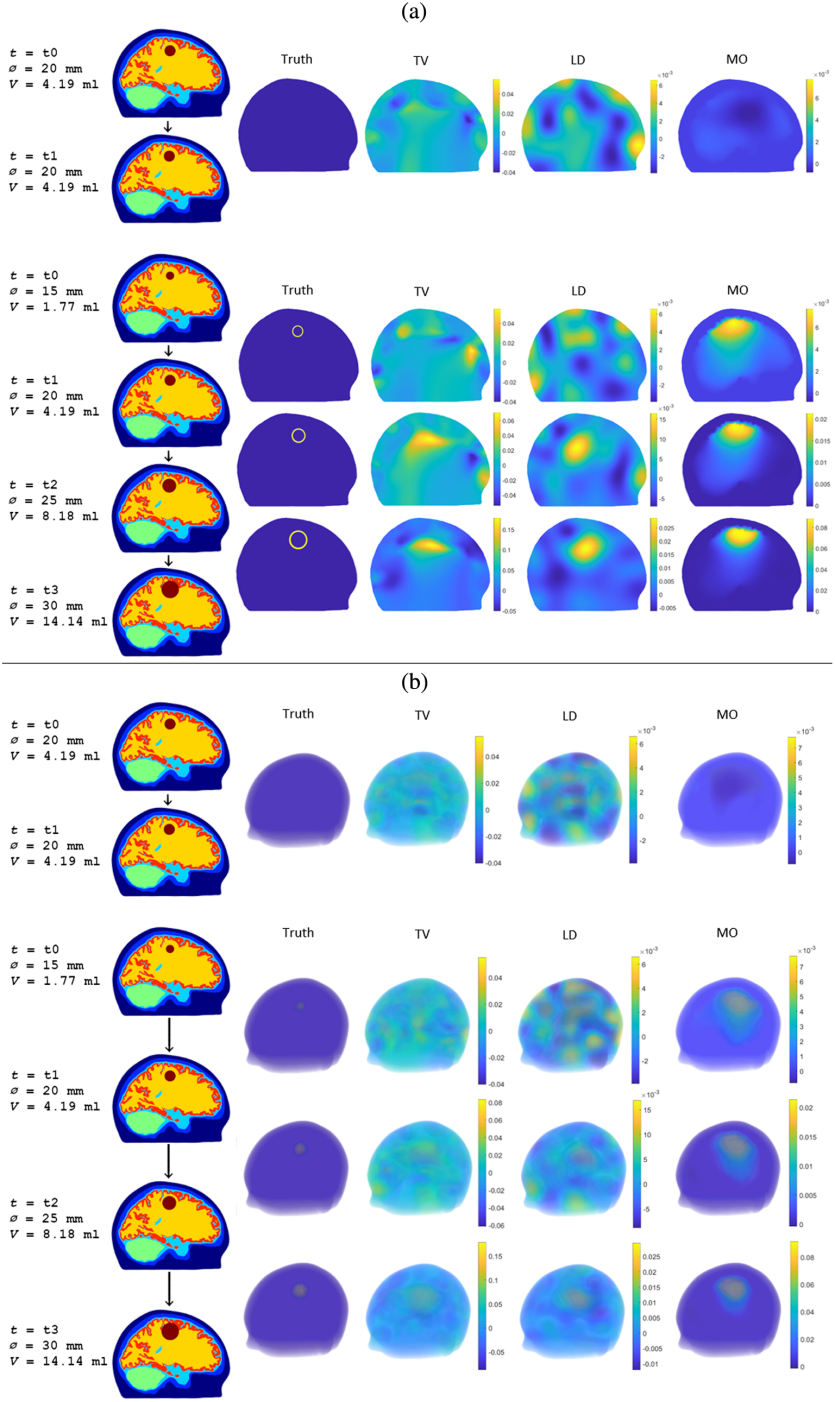
Slices of the computational head model at selected measurement times t corresponding to hemorrhage diameters Ø and hemorrhage volumes V, and estimates of the conductivity change $\delta \sigma =\sigma_{i+1}-\sigma_{i}$ between two consecutive measurement times $t_{i}$ and $t_{i+1}$ for the hemorrhage close to the surface of the brain with the TV regularized absolute imaging algorithm (TV), the linear difference imaging algorithm (LD), and the stroke-monitoring algorithm (MO). (a) Single cross sections through the center of the simulated hemorrhage and (b) transparent 3D plots.

**Fig. 5:**
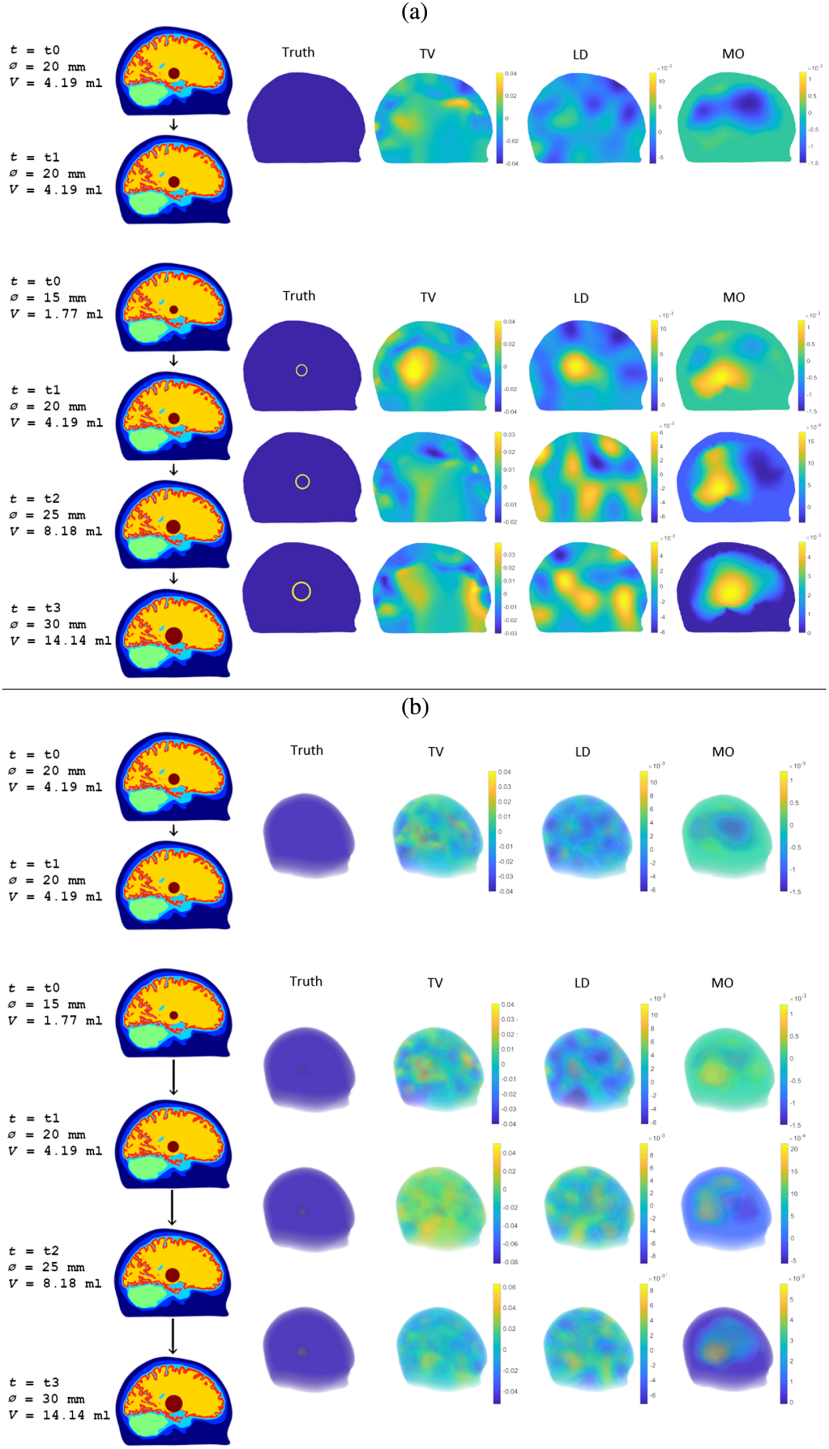
Slices of the computational head model at selected measurement times t corresponding to hemorrhage diameters Ø and hemorrhage volumes V, and estimates of the conductivity change $\delta \sigma =\sigma_{i+1}-\sigma_{i}$ between two consecutive measurement times $t_{i}$ and $t_{i+1}$ for the hemorrhage deep in the basal ganglia region with the TV regularized absolute imaging algorithm (TV), the linear difference imaging algorithm (LD), and the stroke-monitoring algorithm (MO). (a) Single cross sections through the center of the simulated hemorrhage and (b) transparent 3D plots.

The simulated measurement data from all hemorrhage-growth and no-growth scenarios were used to compute estimates of δσ with all three algorithms, TV, LD, and MO. These estimates were computed for both hemorrhage locations, resulting in a total of 126 estimates of δσ. Results for a single hemorrhage progression chain (growth from 15 to 20 to 25 to 30 mm hemorrhage diameter, corresponding to volume increases of 2.42, 3.99, and 5.96 ml) and for a single no-growth case (20 to 20 mm hemorrhage diameter, corresponding to a volume change of 0 ml) are shown in Fig. 4 for the hemorrhage close to the surface of the brain and in Fig. 5 for the hemorrhage deep in the basal ganglia region.

For the cortical hemorrhage close to the surface of the brain, the cross sections in Fig. 4(a) show that all three algorithms are capable of indicating the largest and second largest volume change (fourth and third rows of estimates), but the smallest volume change (second row of estimates) is indicated only by the stroke-monitoring algorithm. Furthermore, an examination of the transparent 3D plots in Fig. 4(b) reveals that the estimates of the second largest volume change (third row of estimates) with the reference algorithms contain additional conductive inclusions that could be misinterpreted as additional hemorrhages. Overall, the estimates produced by the stroke-monitoring algorithm show a much more regular and better-localized conductivity change compared to the estimates of the reference algorithms.

The more challenging hemorrhage location deep in the basal ganglia region causes deterioration of estimate quality for all algorithms as is clearly visible in the cross sections in Fig. 5(a). For the reference algorithms, even the cross sections that seem to indicate the expansion somewhat correctly (second row of estimates), they show just one of many conductive artefacts, as revealed by a closer examination of the transparent 3D plots in Fig. 5(b). Also, the estimates from the stroke-monitoring algorithm are less regular and localized, and now more of the estimates of the conductivity change contain negative values, both indicative of less reliable results. Nevertheless, out of the three algorithms, the estimates of the stroke-monitoring algorithm best indicate the expansion of the hemorrhage also in this more challenging monitoring setup.

**Fig. 6:**
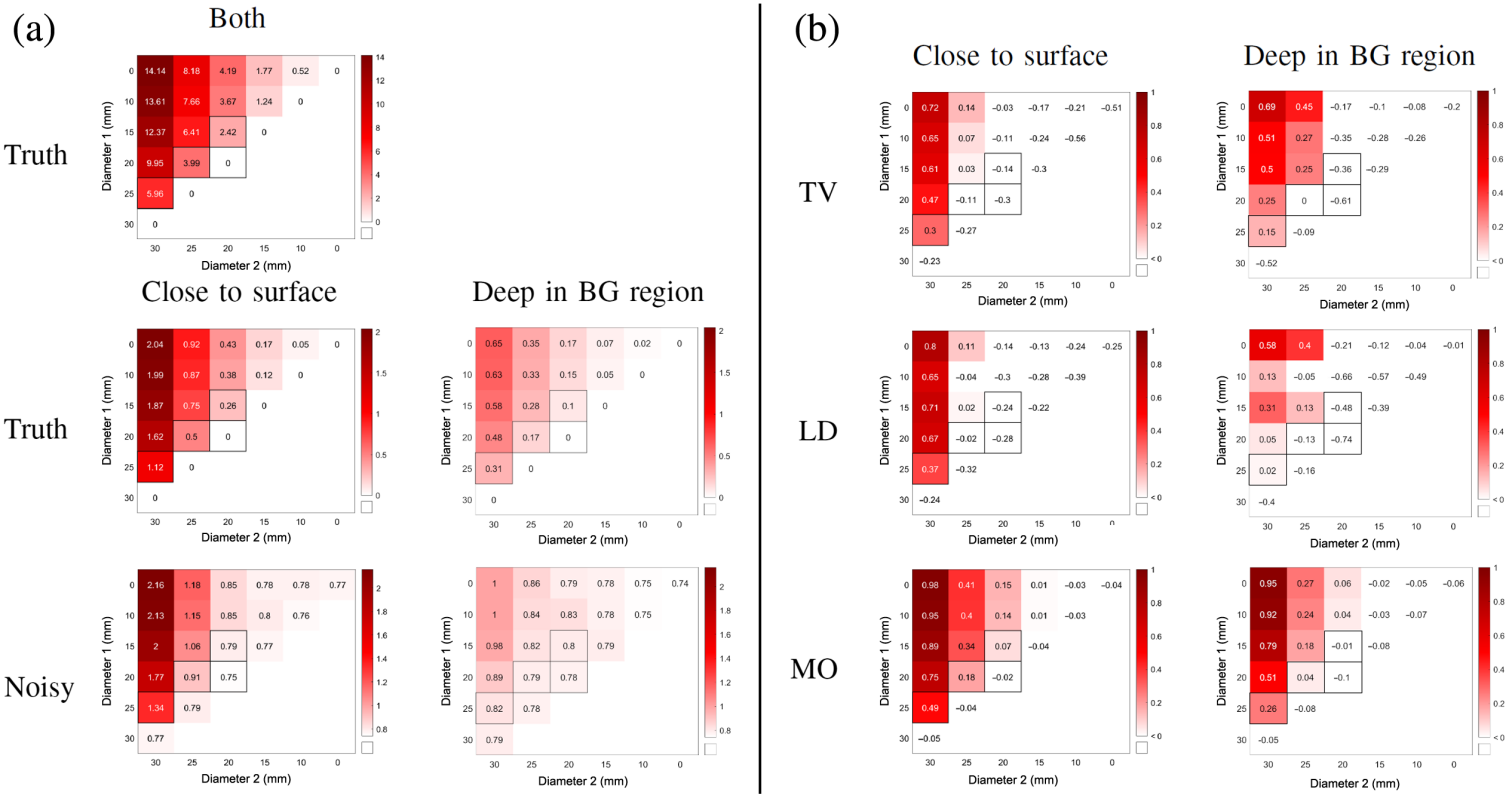
Comparison of all 126 cases. Diameter 1 and 2 are the diameters of the simulated hemorrhage at the time of the first and the second measurements, respectively. (a) First row: the true volume changes in ml; second row: the norms of the noise-free difference data for the two hemorrhage locations in mV; third row: norms of the noisy difference data in mV. (b) The adjusted normalized integrals $Q_{D_{i},D_{j}}$ for the TV regularized absolute imaging algorithm (TV), the linear difference imaging algorithm (LD), and the stroke-monitoring algorithm (MO).

The results for all 126 estimation cases are shown as heat maps in Fig. 6. The first row shows for reference the true volume changes and the second row shows the norms of the noise-free difference data for the two hemorrhage locations. The third row shows the norms of the noisy difference data that show the magnitude of the change in the noisy measurement data from the hemorrhage growth, with the no-growth values showing only the level of the measurement noise. For comparison of the estimates, the integrals $I_{D_{i},D_{j}}=\int_{\Omega }(\delta \sigma {)}_{D_{i},D_{j}}dx$ were computed for all inclusion diameters $D_{i}$ and $D_{j}$ and then normalized for each algorithm and simulated hemorrhage location separately. These were used to compute the adjusted normalized integrals shown in the heat maps

$$Q_{D_{i},D_{j}}=I_{D_{i},D_{j}}-\max(\mathrm{abs}(I_{D_{k},D_{k}})),$$ (31)

where $I_{D_{k},D_{k}}$ are the normalized integrals of the no-growth scenarios, and the maximum of their absolute values is here regarded as a rough approximation for the maximum deviation in $I_{D_{i},D_{j}}$ caused by measurement noise. Removing $I_{D_{k},D_{k}}$ adjusts the normalized integrals so that all resulting $Q_{D_{i},D_{j}}<0$ are expected to correspond to estimates that are comparable to those caused by noise alone and are thus not expected to be useful. This is further highlighted by the chosen color scheme where all $Q_{D_{i},D_{j}}<0$ are shown in white. In contrast, the closer the values are to 1, the higher the expected detectability of hemorrhage growth is.

Overall, the heat maps of the stroke-monitoring algorithm have larger values than either of the reference algorithms, indicating better detectability of hemorrhage growth. The difference is clearest when comparing the second or the third columns in the heat maps. The values highlighted with black borders in the heat maps correspond to the estimates shown in Figs. 4 and 5. The highlighted norms of the noisy difference data show that for the hemorrhage close to the surface of the brain the two larger conductivity changes (20 to 25 and 25 to 30 mm) are clearly different from the no-growth cases, but the value corresponding to the smallest conductivity change (15 to 20 mm) is already very close to the no-growth cases. This small conductivity change was visible only in the estimate from the monitoring algorithm and this is at least partly because the algorithm benefits from utilizing both measurements and their connection, unlike the linear difference algorithm that uses difference data or the TV regularized absolute imaging algorithm that treats the measurements as completely separate.

## 5 Conclusion

In this paper, we used simulated data from an anatomically detailed computational head model to study the feasibility of EIT for continuous bedside monitoring of ICHs and detection of secondary hemorrhages and got promising results using a tailor-made, patient-specific stroke-monitoring algorithm. The monitoring algorithm was referenced against a TV-regularized absolute imaging algorithm and a linear difference imaging algorithm, and it was shown that the proposed stroke-monitoring algorithm is capable of indicating smaller changes in the simulated hemorrhages than either of the reference algorithms. In the simulation tests of this paper, the smallest volume change in the simulated hemorrhage detected by the proposed stroke-monitoring algorithm was 2.42 ml for the cortical hemorrhage close to the surface of the brain and 3.99 ml for the hemorrhage deep in the basal ganglia region.

Further testing of the feasibility of EIT for monitoring of ICHs and detection of secondary hemorrhages is envisioned to include more complex simulation studies, laboratory phantom measurements that build upon the ones presented by Toivanen et al.,^15^ live animal measurements, and eventually measurements from human stroke patients. Future development presents a number of challenges, including the uncertainty of the electrode locations, electrode impedance variations, and effects of computational domain truncation. However, our results provide first simulation-based proof of concept, and we believe that EIT-based bedside stroke monitoring becomes a valuable tool in follow-up of ICH patients for an early warning of changes in the brain tissue of acute stroke patients that would prompt an immediate need for control CT scanning. In the most severe cases, this would lead to timely decision to advance to intensive care and to neurosurgical intervention.

## Data Availability

The codes and the data utilized in this study are not publicly available. The codes and the data may be requested from the authors, and permission for access will be granted or denied on a case-by-case basis.
